# Predictive Effects of Lung function test on Postoperative Pneumonia in Squamous Esophageal Cancer

**DOI:** 10.1038/srep23636

**Published:** 2016-03-23

**Authors:** Ran Wei, Wei Dong, Hongchang Shen, Yang Ni, Tiehong Zhang, Yibing Wang, Jiajun Du

**Affiliations:** 1 Department of Thoracic Surgery, Shandong Provincial Hospital Affiliated to Shandong University, Shandong University, Jinan, 250021, China; 2 Department of Oncology, Shandong Provincial Hospital Affiliated to Shandong University, Shandong University, Jinan, 250021 China; 3 Institute of Oncology, Shandong Provincial Hospital Affiliated to Shandong University, Shandong University, Jinan, 250021, China; 4 Department of Surgery, Shandong Provincial Hospital Affiliated to Shandong University, Jinan, 250021, China

## Abstract

Pulmonary function tests had prospective implications for postoperative pneumonia, which occurred frequently after esophagectomy. Understanding factors that were associated with pulmonary infection may help in patient selection and postoperative management. We performed a retrospective review of 2 independent cohorts including 216 patients who underwent esophagectomy between November 2011 and May 2014, aiming at identifying predictors of primary pneumonia. Univariate analysis was used to identify potential covariates for the development of primary pneumonia. Adjustments for multiple comparisons were made using False Discovery Rate (FDR) (Holm-Bonferroni method). Multivariable logistic regression analysis was used to identify independent predictors and construct a regression model based on a training cohort (n = 166) and then the regression model was validated using an independent cohort (n = 50). It showed that low PEF (hazard ratio 0.97, *P* = 0.009) was independent risk factors for the development of primary pneumonia in multivariate analyses and had a predictive effect for primary pneumonia (AUC = 0.691 and 0.851 for training and validation data set, respectively). Therefore, PEF has clinical value in predicting postoperative pneumonia after esophagectomy and it may serve as an indicator of preoperative lung function training.

Pneumonia was the most common postoperative pulmonary complications for patients with esophagectomy, which has been reported in several literatures^1^,^2^. Many studies have revealed that various factors may lead to pulmonary complications, including advanced age, abnormal chest radiograph^3^, diabetes mellitus^4^, history of major operation and abnormal lung function test^1^, low serum albumin^5^, and postoperative analgesia^6^. However, little work has been done on the prediction of pneumonia, which may be different from other pulmonary complications.

Postoperative pneumonia could be divided into two groups, as we consider them different causes. In some cases, pneumonia occurred after an uneventful operation without any obvious predisposing factor and it is called primary pneumonia. By contrast, some cases are clearly the consequence of a complicated procedure or other postoperative morbidities. These cases were defined as secondary pneumonia. With the development of perioperative management of esophagectomy, the incidence of pneumonia decreased, especially for secondary pneumonia^7^,^8^. Knowledge of which patients are at higher risk for primary pneumonia may contribute to selection of patients for surgical intervention, institution of risk reduction measures, and allocation of resources for postoperative care.

Pulmonary function test has been considered as independent prediction factors of postoperative pulmonary complications^9^ and long-term survival^10^. But investigators put more emphasis on forced expiratory volume in 1 second%(FEV1%), forced vital capacity(FVC)^9^,^11^ and diffusion capacity of the lung for carbon monoxide (DLCO)^11^, little attention has been done on peak expiratory flow (PEF), which may be a more practical physiologic measurement to obtain from physically debilitated or having cognitive impairment elderly persons^12^,^13^. PEF represented the ability to cough effectively and was correlated with the pleural pressure during FVC manoeuvre. In addition, patients with central airway obstruction or upper airway occlusion may have lower PEF without a decrease in FEV1 and /or VC^13^, so normal or higher PEF may be related with decreased likelihood of aspiration of oropharyngeal bacteria into the lower respiratory tract. As individual institution and clinical trials do not have a common system for documenting the occurrence or severity of complications associated with esophagectomy, it may lead to discordance in reporting surgical complications as well as differ in conclusions. We conducted this study to identify factors that were related with postoperative primary pneumonia after esophagectomy according to the proposed system for defining and recording associated with esophagectomy by the Esophagectomy Complications Consensus Group^14^.

## Results

### Clinicopathological characteristics of patients

For the training cohort, patients baseline characteristics and preoperative, intraoperitive risk factors associated with primary pneumonia were shown in Tables 1 and 2. The 166 patients included 131 men (79.17%) and 35 women (20.83%), with a mean age of 61 (range 41–76). The incidence of postoperative primary pneumonia was 10.24% (n = 17). All the primary pneumonia occurred within the first postoperative week, with its peak incidence in the first 3 days. After complete resection, 165 patients (99.40%) were diagnosed with squamous cell carcinoma, 1 (0.60%) with small cell carcinoma. The preoperative and tumor specific variables were compared between the two groups. Weight loss from presenting symptom to surgery (group I, 2.08 ± 2.83; group II, 0.77 ± 1.30, *P* = 0.002), preoperative hemoglobin (group I, 140.71 ± 12.80; group II, 147.65 ± 10.75, *P* = 0.03) and heart disease (group I, 13.42%; group II, 35.29%, *P *= 0.03) between two groups showed significant difference. Unexpected, pathological stage of tumor and existence of diabetes and respiratory comorbidities (including chronic bronchitis, emphysema, chronic obstructive pulmonary disease (COPD) and asthma) were not associated with the occurrence of primary pneumonia. There was significant difference in the percent predicted FEV1 and PEF between the two groups (*P *= 0.02 and *P *= 0.008 respectively). The selection of surgical approaches and blood loss did not affect the incidence of pulmonary infection.

The postoperative managements were shown in Table 3. The ICU stay, duration of oxygen supplement and respirator use did not have significant differences between the two groups as well. By contrast, the use of antibiotics was significantly longer in group II (*P *= 0.04).

### Construction of logistic regression model

**Figure 1:**
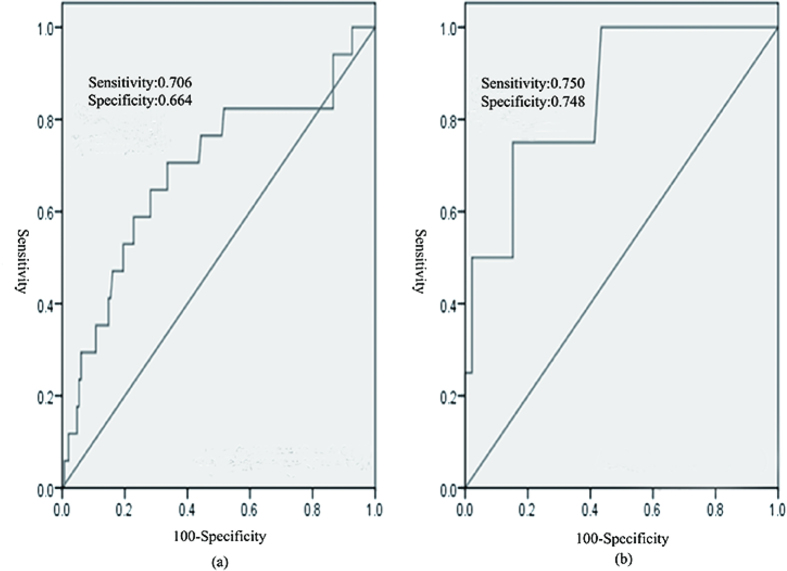
Receiver operating characteristic curve analysis for primary pneumonia after esophagectomy. Area under the curve (AUC) estimation for the peak expiratory flow (PEF) in (**a**) the training set, (**b**) the validation set.

Among the aforementioned preoperative and intraoperative risk factors, only weight loss and PEF remained statistically significant after adjusting for FDR (*P*-value = 0.078 and 0.156 respectively). These two factors were incorporated into the logistic regression analyses (Table 4). It showed that low PEF (hazard ratio 0.97, *P *= 0.009) was independent risk factors for the development of primary pneumonia in multivariate analyses. The predicted probability of being diagnosed with primary pneumonia from the logistic model based on the percent predicted PEF, logit (*P* = primary pneumonia) = 0.061−0.034*PEF. The predicted performance for the established PEF logistic model was evaluated by using ROC analysis in Fig. 1(a) (AUC 0.691; 95%CI, 0.543 to 0.839; sensitivity 70.6%, specificity 66.4%).

### Validation of predictive accuracy of the logistic regression model

There was no significant difference in the distribution of baseline characteristics and perioperitive factors between the training and validation data sets. The detail information could be found as Supplementary Table S1 online. The parameters estimated from the training data set were used to predict the probability of being diagnosed with primary pneumonia for the independent validation cohort (n = 50). Similarly, the predicted probability was used to construct the ROC curve (Fig. 1(b)). The analysis demonstrated that the PEF had high accuracy in discriminating patients with higher risk of having primary pneumonia from non-pneumonia patients after esophagectomy (AUC0.851; 95% CI, 0.000 to 1.000; sensitivity 75.0%, specificity 74.8%).

## Discussion

In our study, we only focused on the predictive factors of primary pneumonia, which occurred directly after an uneventful operation^1^. The frequency of pneumonia was 11.31% which compare favorably with those previously reported in the literatures^15^. Of note, 2 patients of them were secondary to anastomotic leakage, with only 17 (10.12%) patients having primary pneumonia. We found that primary pneumonia was not significantly related with patients’ most comorbidities, such as diabetes, respiratory disease and major surgery, which was beyond our expectation. The improvements in the preoperative and postoperative management of comorbidities and selection bias may be partly responsible for the lack of significant association. Most of patients with diabetes would be sent to SICU after the surgery for higher risk of undergoing severe complications. In addition, preoperative tobacco cessation and lung function training may also have contributed to the reduction of pneumonia. There was no significant association between pneumonia and hospital stay, ICU stay, and respirator use, except for antibiotic use. It may be ascribed to the early occurrence of primary pneumonia (within the first postoperative week) and utility of sufficient sensitivity antibiotics, which prevented most patients with pneumonia from getting so worse that decay patients leaving hospital. Furthermore, some other severe complications, such as respiratory failure, ARDS and anastomotic leakage, substantially affected rehabilitation of postoperative patients, duration of respirator use and length of hospital stay.

As a measure of lung function, PEF was an independent predicted factor for the occurrence of primary pneumonia in our study. Although lower percent predicted FEV1, PEF, weight loss and hemoglobin were identified as possible risk factors for primary pneumonia by univariate analyses (p < 0.05), only percent predicted PEF and weight loss had predictive value after FDR correction (FDR P value ≤ 0.20). Furthermore, PEF was the only risk factor of primary pneumonia in multivariate analysis. It seemed that PEF had more applicability to patients with esophagectomy in predicting postoperative primary pneumonia. Furthermore, it was validated in an independent series of patients with esophagectomy.

Various factors that have been shown to be correlated with pulmonary complications after esophagectomy cannot easily be altered preoperatively in order to reduce the frequency of such complications. For example, D’Journo *et al*. and Akutsu *et al*. have demonstrated that the contamination of the oropharynx or tracheobronchial tree, or both, is likely to be a source of postoperative pneumonia^16^,^17^. Their findings suggested that appropriate use of perioperative antibiotics and an improved oral hygiene could reduce the incidence of postoperative pneumonia. In addition, it was intuitively likely to reduce complications through improving preoperative general physical fitness of a patient, although it cannot easily be accomplished in a short period before the operation^17^. Focusing on lung function improving rather than promoting overall fitness may offer similar risk reduction in the allotted time frame. Some studies have shown the effects of preoperative pulmonary function training on the occurrence of pneumonia after esophagectomy^18^,^19^. Meanwhile, some clinical trials were on the way (NCT01893008 and NCT01766349). The conclusions were still in controversial. Our study showed that the PEF may serve as a better indicator of pulmonary function training program to predict the possibility of primary pneumonia which accorded with the results of Agrelli *et al*^19^. Recent publications have found that lung function training could increase PEF of smokers, which may be helpful for patients planning to undergo esophagectomy^20^.

However, variations in approaches to esophagectomy, postoperative management techniques, and increasing use of neoadjuvant therapy may affect our conclusion to some extent. In addition, spirometers were usually unsuited for measuring PEF compared with PEF meters, which may lead to bias. Therefore, more randomized controlled trials are warranted to test whether evaluation of PEF is applicable to patients who are undergoing esophagectomy.

## Patients and Methods

### Patients

In training cohort, 174 patients who underwent esophagectomy from November 2011 to October 2013 at Shandong Provincial Hospital Affiliated to Shandong University were included in our study. 4 Patients without preoperative lung function test records as well as 2 patients who underwent concomitant pulmonary lobectomy were excluded. In addition, 2 patients who were diagnosed with secondary pneumonia after esophagectomy were not incorporated into our analyses for its different etiology. In validation cohort, a series of 50 new patients who underwent esophagectomy in the same hospital between October 2013 and May 2014 was used to validate the model which was constructed in the training phase. The study protocol was approved by the Ethics Committee of Shandong Provincial Hospital. The methods were carried out in accordance with the approved guidelines. Written information was provided and informed consent was obtained from all subjects.

All patients in the two independent cohorts had basic hematological and biochemical tests, lung function tests, abdomen B ultrasonography, and ultrasound cardiography, chest radiograph, and electrocardiograph, gastroscopy, barium contrast study taken as preoperative evaluation and diagnostic procedures. Operations included open transthoracic and hybrid minimally invasive esophagectomy (MIE). Hybrid MIE was defined as a surgery performed by either a laparoscopic or thoracoscopic technique with concomitant open surgery. The McKeown, Ivore-Lewis and Sweet approaches were choosed, depending on the location of the tumor and the physical status of patients. Reconstruction of upper gastrointestinal continuity was usually restored with a gastric conduit placed in the orthotopic route (after Ivor-lewis and Mckeown approaches), while the retrosternal route was chosen in the Sweet approach. All surgical procedures were performed by the same surgical team. Prophylactic cephalosporin was given half an hour before operation and every 3 hours during surgery. Right bronchial artery and thoracic duct would be preserved as much as possible in cases without suspected direct invasion.

Preventive use of antibiotics was allowed for the first 24 hours postoperatively and even 48 hours for patients with high risk of pulmonary complications, unless they had been judged to have pneumonia according to American Thoracic Society (ATS) guidelines^21^. All patients with suspected pneumonia would be collected endotracheal sputum to be cultured with or without gram stain of a deep respiratory tract culture, and then received initial empiric antibiotic treatment. Therapy would be modified by day 2 or 3 according to the clinical responses and sputum culture results. All patients were routinely given supplemental oxygen as well as analgesia, intravenous fluid administration for the first few days. Intravenous albumin supplement was given for those whose albumin concentration were less than 35 g/L. Suction of sputum through cricothyroid membrane puncture was administered to aid expectoration of retained sputum and secretion. Oxygen saturation was routinely checked every 3 hours during the first few days postoperatively as well. Oral intake was begun after barium esophagogram had ruled out anastomotic leakage. Patients would leave the hospital when they had begun oral intake for several days without existence of any abnormal symptoms or they want to convalesce at an offsite medical facility for socioeconomic reasons. Follow-up was carried out at regular intervals by a dedicated team, with the first time on the thirtieth day after the surgery. The occurrence of any complications, readmissions, mortality within 30 days or 90 days, and metastasis or recurrence, death time and cause of death after discharge were recorded.

### Data collection

The medical records of the all patients (216 patients) were retrospectively collected according to the proposed system for defining and recording associated with esophagectomy by the Esophagectomy Complications Consensus Group^14^. Collected data included demographics, preoperative and intraoperative characteristics. Patients who were current smokers or whose smoking indexes were more than 400 were defined as ever-smokers. Heart disease included heart valve disease and coronary heart disease, while respiratory disease included chronic bronchitis, emphysema, chronic obstructive pulmonary disease (COPD) and asthma. Lung function test was performed using a MasterScreen Body Jaeger spirometer (CareFusion Ltd, Viasys Healthcare, Hoöchberg, Germany). Spirometric reference equations are derived from the study conducted by the China’s Ministry of Health which included large groups of Chinese healthy adults in 1988, and this reference equation had been proven to be suitable at present^22^. Pneumonia was diagnosed using chest radiograph plus at least two clinical evidence (fever greater than 38, leukocytosis or leukopenia, and purulent secretions) according to American Thoracic Society and Infectious Diseases Society of America^21^. It was categorized as primary and secondary, because secondary pneumonia was more associated with aspiration or anastomotic leakage after the operation^1^. Physicians distinguished the primary pneumonia from secondary pneumonia according to the existence of these obvious predisposing factors. Tumor-specific variables included the level of tumor and pTNM stage followed the seventh edition of the American Joint Committee on Cancer TNM staging^23^.

### Statistical Analysis

We evaluate the potential perioperative risk factors associated with primary pneumonia in univariate analysis. Continuous variables were expressed as mean ± standard deviation with evaluated by Student’s t test, and categoric variables were presented as frequency and percentages with analyzed by X^2^ test or Fisher’s exact test when appropriate. Adjustments for multiple comparisons were made using False Discovery Rate (FDR) (Holm-Bonferroni method). Risk factors were considered significant if the FDR *P*-value ≤0.20 and they would be incorporated into the logistic regression analysis. Multivariate analyses of risk factors for pneumonia were carried out with logistic regression using forward selection algorithm. The parameters of the logistic model from the training phase were applied to an independent cohort of 50 patients for validating the diagnostic performance of the selected risk factors. In these analyses, all probabilities were two-tailed and *P* values less than 0.05 were regarded as statistically significant. All statistical analyses were performed using the SPSS (version 17.0, SPSS Inc., Chicago, IL).

## Additional Information

**How to cite this article**: Wei, R. *et al*. Predictive Effects of Lung function test on Postoperative Pneumonia in Squamous Esophageal Cancer. *Sci. Rep.* **6**, 23636; doi: 10.1038/srep23636 (2016).

## Supplementary Material

Supplementary Information

## Figures and Tables

**Table 1:** Baseline patient characteristics and preoperative risk factors for patients undergoing esophagectomy.

Viariable a	All patients N = 166	Group I N = 149	Group II N = 17	*P* value
Age	60.77 ± 7.66	60.97 ± 7.61	58.94 ± 8.01	0.30
Male	131(78.92%)	116(77.85%)	15(88.24%)	0.53
Body mass index (kg/m^2^)	22.95 ± 3.24	22.98 ± 3.34	22.61 ± 2.24	0.54
Medical history, n (%)				
Hypertension	41(24.70%)	36(24.16%)	5(29.41%)	0.77
Diabetes mellitus	16(9.64%)	15(10.07%)	1(5.88%)	1.00
Respiratory	69(41.57%)	61(40.94%)	8(47.06%)	0.80
Heart disease	26(15.66%)	20(13.42%)	6(35.29%)	0.03
Gastrointestinal ulcer	124(75.90%)	110(73.83%)	14(82.35%)	0.57
Cerebrovescular disease	4(2.41%)	3(2.01%)	1(5.88%)	0.35
Major surgery	13(7.83%)	12(8.05%)	1(5.88%)	1.00
Duration of presenting symptoms(months)	3.66 ± 7.83	3.36 ± 7.25	6.28 ± 11.71	0.33
Time between diagnosis and surgery (days)	6.80 ± 3.17	6.65 ± 2.69	8.12 ± 5.89	0.32
Weight loss (kg)	1.95 ± 2.74	2.08 ± 2.83	0.77 ± 1.30	0.002
Albumin(g/L)	41.49 ± 2.90	41.36 ± 2.93	42.62 ± 2.36	0.09
Lymphocyte number≥1500( /mm^2^)	107(64.46%)	97(65.10%)	10(58.82%)	0.60
Hemoglobin(mg/dL)	141.43 ± 12.75	140.71 ± 12.80	147.65 ± 10.75	0.03
Smoking history (ever-smokers)	92(55.42%)	82(55.03%)	10(58.82%)	1.00
Tumor localization, n (%)				0.11
Upper	15(9.04%)	11(7.38%)	4(23.53%)	
Middle	57(34.34%)	52(34.90%)	5(29.41%)	
Lower	94(56.62%)	86(57.72%)	8(47.06%)	
Pulmonary function test				
VC	93.79 ± 16.57	94.66 ± 16.73	87.54 ± 13.12	0.09
FVC	89.86 ± 15.30	90.71 ± 15.07	83.39 ± 15.52	0.06
FEV1	95.79 ± 18.31	97.09 ± 17.94	86.18 ± 18.61	0.02
MVV	87.91 ± 19.65	88.67 ± 18.96	79.60 ± 24.09	0.07
PEF	88.81 ± 21.25	90.18 ± 20.74	75.67 ± 22.91	0.008
DLCO	82.46 ± 15.61	82.29 ± 15.73	84.41 ± 15.70	0.59

^a^data are shown as mean  ±  standard deviation or n (%) as appropriate. VC: vital capacity; FVC: forced vital capacity; FEV1: forced expiratory volume in 1 second; MVV: maximum ventilator volume; PEF: peak expiratory flow; DLCO: diffusing capacity for carbon monoxide.

**Table 2:** Operative risk factors for patients undergoing esophagectomy.

Viariables^a^	All-patients N = 166	Group I N = 149	Group II N = 17	*P* value
Type of esophagectomy, n (%)				0.44
Sweet	94(56.63%)	85(57.05%)	9(52.94%)	
Ivor-Lewis	42(25.30%)	39(26.17%)	3(17.65%)	
Mckeown	30(18.07%)	25(16.78%)	5(29.41%)	
Site of anastomosis				0.33
Neck	39(23.49%)	27(18.12%)	12(70.59%)	
Chest	127(76.51%)	122(81.88%)	5(29.41%)	
Route of reconstruction				0.80
Retrosternal	94(56.63%)	85(57.05%)	9(52.94%)	
Orthotopic	72(43.37%)	64(42.95%)	8(47.06%)	
Thoracic duct ligation	45(27.11%)	38(25.50%)	7(41.18%)	0.25
Blood loss (ml)	249.82 ± 165.60	247.11 ± 162.65	276.67 ± 187.91	0.51
Intraoperative blood transfusion (ml)	60.54 ± 119.06	58.72 ± 117.08	76.47 ± 138.20	0.56
Intraoperative erythrocyte transfusion (U)	0.54 ± 1.13	0.51 ± 1.10	0.82 ± 1.42	0.26
Vital volum/weight (L/kg)	7.24 ± 1.24	7.21 ± 1.27	7.47 ± 0.97	0.44
Length of anesthesia (min)	277.14 ± 89.76	274.70 ± 88.95	298.53 ± 96.69	0.30
Length of operation (min)	240.24 ± 86.42	237.55 ± 84.76	263.82 ± 99.55	0.24
Tumor length (cm)	4.79 ± 2.07	4.81 ± 2.08	4.65 ±± 2.03	0.76
American society of Anesthesiologist(ASA) score				0.11
I	14(8.43%)	14(9.40%)	0(0.00%)	
II	139(83.74%)	125(83.89%)	14(82.35%)	
III	13(7.83%)	10(6.71%)	3(17.65%)	
T				0.72
Tis	5(3.01)	5(3.36%)	0(0.00%)	
T1	10(6.02%)	9(6.04%)	1(5.88%)	
T2	27(16.27%)	25(16.78%)	2(11.77%)	
T3	120(72.29%)	107(71.81%)	13(76.47%)	
T4	4(2.41%)	3(2.01%)	1(2.13%)	
N				0.46
N0	87(52.41%)	79(53.02%)	8(47.06%)	
N1	38(22.89%)	32(21.48%)	6(3.53%)	
N2	28(16.87%)	25(16.78%)	3(17.65%)	
N3	13(7.83%)	13(8.72%)	0(0.00%)	
Stage				0.88
0	5(3.01%)	5(3.36%)	0(0.00%)	
I	9(5.42%)	8(5.37%)	1(5.88%)	
II	78(46.99%)	71(47.65%)	7(41.18%)	
III	74(44.58%)	65(43.62%)	9(52.94%)	

^a^data are shown as mean ± standard deviation or n(%) as appropriate.

**Table 3:** Postoperative data of patients undergoing esophagectomy.

Viariable^a^	All patients N = 166	Group I N = 149	Group II N = 17	*P*value
Blood transfusion (ml)	231.52 ± 362.80	230.41 ± 373.88	241.18 ± 254.48	0.91
Erythrocyte transfusion (U)	0.49 ± 1.13	0.44 ± 1.03	0.94 ± 1.75	0.26
ICU (days)	0.31 ± 1.67	0.27 ± 1.61	0.71 ± 2.17	0.31
Duration of respirator use (days)	0.13 ± 0.68	0.07 ± 0.26	0.65 ± 1.94	0.24
Oxygen inhalation (days)	9.17 ± 8.32	9.19 ± 8.64	9.06 ± 4.87	0.95
Analgesic (days)	5.36 ± 2.39	5.39 ± 2.44	5.12 ± 1.93	0.66
Length of stay (days)	27.28 ± 18.10	26.38 ± 16.75	35.18 ± 26.64	0.20
Antibiotics (days)	5.11 ± 5.01	4.83 ± 4.84	7.53 ± 5.94	0.04

^a^data are shown as mean ± standard deviation or n(%) as appropriate.

**Table 4:** Multivariate analysis of primary pneumonia.

Risk Factors	Univariate Analysis (p value)	Multivariate Analysis (p value)	Hazard Ratio (95% confidence range)
PEF	0.008	0.009	0.97(0.94−0.99)
Weight loss	0.002	0.10	

PEF: peak expiratory flow.
