# Supplementary material for: Predictive Effects of Lung function test on Postoperative Pneumonia in Squamous Esophageal Cancer
Source: Sci Rep. 2016 Mar 23;6:23636. doi: 10.1038/srep23636 (PMC4804297; doi:10.1038/srep23636)
Supplement: Supplementary Information [file srep23636-s1.pdf]

Supplementary Information

Predictive Effects of Lung function test on Postoperative Pneumonia in Squamous Esophageal Cancer

Ran Wei[1,3*], Wei Dong[1*], Hongchang Shen[2], Yang Ni[2], Tiehong Zhang[2], Yibing Wang[4&], Jiajun Du[1,3&]

[1] Department of Thoracic Surgery, Shandong Provincial Hospital Affiliated to Shandong University, Shandong University, Jinan, 250021， China;

[2]Department of Oncology, Shandong Provincial Hospital Affiliated to Shandong University, Shandong University, Jinan, 250021 China;

[3] Institute of Oncology, Shandong Provincial Hospital Affiliated to Shandong University, Shandong University, Jinan, 250021, China;

[4]Department of Surgery, Shandong Provincial Hospital Affiliated to Shandong University, Jinan, 250021, China.

[&]Corresponding author: Yibing Wang(wyb0616@163.com) and Jiajun Du (dujiajun@sdu.edu.cn)

*These authors contributed equally to this work.

**Viariable** [a]	**Training N=166**	**Validation N=50**	***P* value**
Age	60.77±7.66	58.84±7.41	0.12
Male	131(78.92%)	38(76.00%)	0.70
Body mass index (kg/m^2)	22.95±3.24	23.96±3.29	0.06
Medical history, n (%)			
Hypertension	41(24.70%)	12(24.00%)	1.00
Diabetes mellitus	16(9.64%)	4(8.00%)	1.00
Respiratory	69(41.57%)	20(40.00%)	0.87
Heart disease	26(15.66%)	9(18.00%)	0.83
Major surgery	13(7.83%)	8(16%)	0.18
Type of esophagectomy, n (%)			0.57
Sweet	94(56.63%)	29(58.00%)	
Ivor-Lewis	42(25.30%)	15(30.00%)	
Mckeown	30(18.07%)	6(12.00%)	
Smoking history (ever-smokers)	92(55.42%)	11(55.03%)	0.71
Length of operation(min)	77.14±89.76	237.60±67.18	0.84
Blood loss(ml)	249.82±165.60	210.20±110.86	0.12
Tumor localization, n (%)			0.80
Upper	15(9.04%)	3(6.00%)	
Middle	57(34.34%)	26(52.00%)	
Lower	94(56.62%)	21(42.00%)	
Stage			0.82
I	5(3.01%)	2(4.00%)	
II	9(5.42%)	23(46.00%)	
III	78(46.99%)	25(50.00%)	
IV	74(44.58%)	0(0.00%)	

Supplementary Table S1. Characteristics of study participants in the training and validation data sets. [a] Continuous data are shown as mean ± standard deviation; categoric data as number (%).
